# Regular Polymeric Microspheres with Highly Developed Internal Structure and Remarkable Thermal Stability

**DOI:** 10.3390/ma14092240

**Published:** 2021-04-27

**Authors:** Małgorzata Maciejewska, Barbara Gawdzik, Magdalena Rogulska

**Affiliations:** Department of Polymer Chemistry, Faculty of Chemistry, Institute of Chemical Sciences, Maria Curie-Skłodowska University in Lublin, Gliniana 33, 20-614 Lublin, Poland; mrogulska@umcs.lublin.pl

**Keywords:** porous microspheres, internal structure, thermal resistance

## Abstract

In this study, the synthesis and characterization of permanently porous polymeric microspheres was presented. The microspheres were obtained via suspension polymerization using diverse functional monomers, such as 4,4′-bis(methacryloyloxymethylphenyl)sulphone, 1,4-bis(methacryloyloxymethyl)benzene, 4,4′-bis(methacryloyloxymethylphenyl)methane, *N*-vinylpyrrolidone, ethylene glycol dimethacrylate, and divinylbenzene as a co-monomer. As porogenic solvents, toluene and chlorobenzene were applied. The main aim of the research was to synthesize polymers having a highly developed internal structure and a good thermal stability. The synthesized materials were characterized by ATR-FTIR, scanning electron microscopy, a size distribution analysis, a low-temperature nitrogen adsorption–desorption method, differential scanning calorimetry, and thermogravimetry coupled with FTIR and inverse gas chromatography. It was found that, depending on the functional monomer, regular microspheres with a specific surface area in the range of 418–746 m^2^/g can be successfully synthesized. Moreover, all the synthesized copolymers showed a good thermal stability. In helium, they exhibited 5% mass losses at temperatures over 300 °C, whereas in air these values were only slightly lower. In addition, the presence of miscellaneous functional groups promoted diverse kinds of interactions. Therefore, the microspheres can be possibly use in many adsorption techniques including high temperature processes.

## 1. Introduction

Permanently porous polymers are a broad class of highly cross-linked, three-dimensional networks that have gained remarkable attention because of their superior inherent porosity, excellent stability, pre-designable and tunable structures. They have been productively exploited as advantageous adsorbents in gas storage and separation [1,2,3,4,5], heterogeneous catalysis [6], energy storage [7,8,9], detection and removal of pollutants from water and other liquids [10,11,12], solid phase extraction [13,14,15], various kinds of chromatography [16,17,18,19,20,21] and other essential functions. Their porous structure, pore size, specific surface areas and application can be directly designed and easily tuned by introducing specific functional building blocks. The functional porous polymers can be created to show stimulus-responsive features capable of reversibly changing the pore structure or even switching between an open and closed porous state after exposure to environmental changes [22]. Such distinct properties are not usually found in other porous materials. Additionally, porous polymers are stable in the whole pH range and their frameworks are composed of light elements (C, H, N, O) and linked with strong covalent bonds. Their organic nature provides a weight advantage compared with other porous materials, among which are silicas, zeolites, metal oxides, and metal-organic frameworks (MOFs). Furthermore, different components can be put together in one single particle to form binary hybrid materials. Polymers can be combined in two main ways: encapsulating one component with another or by placing two components side by side. The former can be classified as a core–shell structure, whereas the latter as a Janus (two-faced) structure. Core–shell nanostructures are a two-component system consisting of an inner layer and a guest nanoparticle of different material that the inner layer encapsulates. These nanostructures are widely used in a variety of applications, such as drug delivery systems [23,24], tribology [25] and smart farming [26]. An interesting form of a core-shell particle is a porous hollow polymeric capsule. Recently, biocompatible, fluorescent and targeting agent-conjugated capsules have been presented. It was shown that they permit a more accurate delivery of anticancer drugs and have a better efficacy for drug accumulation [27]. Similarly, Janus nanoparticles are impressive research materials as they have asymmetric structures and different properties. They are composed of two or more parts that show a discrepancy in their physico-chemical properties and these parts are often contrary in nature [28]. This phenomenon facilitates their applications in drug delivery systems where a single material with several domains can concurrently realize multidrug loading and work as an perfect carrier [29,30]. The anisotropic structure of Janus particles also makes them an ideal choice for theranostics [31].

As demonstrated, the field of high-surface-area materials is extremely diversified. An important group of permanently porous polymers, still extensively explored, remains a porous copolymer of styrene with divinylbenzene (St-*co*-DVB) [32,33,34,35,36]. Because of its hydrophobic nature, St-*co*-DVB interacts with analytes mainly through van der Waals forces and π–π interactions of the aromatic rings that make up the sorbent structure. These kinds of interactions are advantageous in the case of hydrophobic adsorbates but are insufficient in the case of polar ones. In order to improve adsorption of polar analytes, it is necessary to enable their interaction with the sorbent surface through polar interactions. This purpose can be achieved via two main routes: chemical modification of the hydrophobic polymer skeleton by a suitable polar moiety [37,38,39] or by the direct synthesis of polar adsorbents from an appropriate ratio of a hydrophilic monomer and a cross-linker [40,41,42]. It should be emphasized that an effective sorbent with both a high capacity and a fast adsorption rate should possess two principal attributes: functional groups and a highly developed internal structure. However, a frequent phenomenon is the diminution of surface area along with the incorporation of a functional group into the polymer matrix. For example, using a dimethacrylate derivative of naphthalene-2,3-diol as a functional monomer to copolymerize DVB results in a material with a specific surface area of only 72 m^2^/g [43]. An analogical reaction of a diacrylate derivative of 4,4′-sulfanediyldiphenol gives a copolymer with an area equal to 103 m^2^/g [44]. In an equimolar reaction with DVB, 2,3-epoxypropyl methacrylate, an important polar monomer used in numerous syntheses, gives a product with a specific surface area of 143 m^2^/g [45]. For porous polymeric microspheres, it is also important that the porous structure be formed by pores of appropriate diameters. According to IUPAC [46], three classes of pores can be distinguished: micropores (linear dimension below 2 nm), mezopores (dimension 2–50 nm) and macropores (dimension above 50 nm). In many application mezoporous adsorbents are the best choice. The presence of mezopores in the internal structure results in moderate values of specific surface area. Although it is possible to synthesize porous polymers that have extremely high specific surface area, in this case the internal structure is microporous [47]. This fact significantly limits their application spectrum. As can be seen, for new sorbents with functional groups a considerable specific surface area and mesoporous structure are still required.

Another important feature of porous polymers is their thermal behavior. In many of the aforementioned applications, materials with a considerable thermal resistance are indispensable. For example, gas chromatography or catalysis processes are often conducted at temperatures exceeding 200 °C. To ensure substantial thermal stability, the polymers have to be highly cross-linked. For this purpose, both aliphatic and aromatic multifunctional monomers, can be successfully employed. The use of bifunctional glycidyl methacrylate (GMA) with a tetrafunctional cross-linking monomer, i.e., ethylene glycol dimethacrylate (EGDMA) [37,48,49] as well as bifunctional 4-vinylpyridine [50] or GMA [38] with hexafunctional trimethylolpropane trimethacrylate as a cross-linking agent resulted in porous microspheres resistant to about 200 °C. The improvement in thermal stability was obtained by replacing the aliphatic cross-linker, i.e., EGDMA with the aromatic one, i.e., di(methacryloyloxymethyl)naphthalene [51] or by using two tetrafunctional monomers, such as DVB and (4,6-dimethyl-1,3-phenylene)dimethylene bis(2-methylprop-2-enoate) [52] or DVB and dimethacrylate derivatives of naphthalene and diphenylmethanone [43].

The aim of this study was to conduct a detailed investigation into the properties of polymeric microspheres having a highly developed internal structure, remarkable thermal stability and diverse functional group. To achieve this goal, NVP, EGDMA, 4,4′-bis(methacryloyloxymethylphenyl)sulphone (BMPS), 4,4′-bis(methacryloyloxymethylphenyl)methane (BMPM) and 1,4-bis(methacryloyloxymethyl)benzene (BMB) were used as functional monomers. In turn, DVB was used as a co-monomer. Moreover, a St-*co*-DVB copolymer was synthesized and regarded as a reference material. All of the obtained microspheres were comprehensively characterized. Their chemical structure was confirmed using ATR-FTI while their size and porous structure were determined by Mastersized Analyser scanning electron microscopy and a low-temperature nitrogen adsorption–desorption method. The thermal behavior of the copolymeric microspheres, including the identification of volatile decomposition products, was examined by thermogravimetry coupled with FTIR and differential scanning calorimetry. In the paper, the results obtained from inverse gas chromatography are also given.

## 2. Materials and Methods

### 2.1. Chemicals

Divinylbenzene (containing 65 wt% of DVB isomers and ~35 wt% of ethylstyrene) and EGDMA (97.5%) from Merck (Darmstadt, Germany), NVP (99%) from Sigma-Aldrich (Schnelldorf, Germany) and ST (98.5%) from POCh (Poland) were washed with 5% aqueous sodium hydroxide to remove inhibitors. Poly(vinyl alcohol) (PVA) and 2,2′-azoisobutyronitrile (AIBN) purchased from Fluka (Buchs, Switzerland) were used without purification. Toluene, benzene, chlorobenzene, acetone, methanol, butan-1-ol, pentan-2-one, and sodium hydroxide (reagent grade) were obtained from POCh (Poland). BMB [53], BMPS [54] and BMPM [55] were obtained from our laboratory according to the procedures described earlier.

### 2.2. Synthesis of Permanently Porous Microspheres

The copolymerization reaction was conducted in an aqueous suspension medium. Throughout the experiment, 195 mL of distilled water and 6.5 g of PVA were stirred for 6 h at 80 °C in a three-necked flask fitted with a stirrer, water condenser and thermometer. PVA was used as a suspension stabilizer because it permits microspheres with diameters about 100 µm to be obtained. After dissolving PVA, the 15 g organic solution of the monomers (DVB with a comonomer) and 0.075 g of an initiator (AIBN) in 22.5 mL of the diluents was prepared and added while stirring into the aqueous medium. The molar ratio of DVB to a comonomer was kept at 1:1, except for DVB-*co*-BMB copolymer, where the molar ratio was 3:1. When this copolymer was synthesized, the lower amount of DVB resulted in an irregular product shape instead of the desired regular microspheres. Moreover, to obtain a highly porous structure for the resultant copolymeric beads different diluents (toluene and chlorobenzene) were applied. Table 1 presents designations and experimental parameters of the copolymeric microspheres synthesis.

Copolymerization was performed for 20 h at 80 °C. Porous microspheres that formed in this process were filtered, washed with hot water and extracted in a Soxhlet apparatus with acetone, toluene, and methanol. The purified beads were dried under reduced pressure at 60 °C.

### 2.3. Measurement Methods

Attenuated total reflectance Fourier transform infrared (ATR-FTIR) spectra were obtained with a Bruker Tensor 27 FTIR spectrometer (Ettlingen, Germany). The FTIR spectra were recorded in the 4000–600 cm^−1^ spectral range with 32 scans per spectrum at a resolution of 4 cm^−1^.

The textural characterization of the investigated samples was determined on the basis of the low-temperature nitrogen adsorption/desorption method. Nitrogen adsorption/desorption isotherms at −196 °C were determined volumetrically using ASAP 2405N analyzer (Micromeritics Corp., Norcross, GA, USA). The measurements of the porous structure of the copolymers were preceded by outgassing (10^−2^ mm Hg) of the samples at 140 °C for 2 h. To calculate the main parameters of the porous structure the Brunauer–Emmet–Teller (BET) method was applied. The linear BET plots were used to evaluate the specific surface area (*S_BET_*). The total pore volume (*V*) was estimated from a single point adsorption at a relative pressure *p*/*po* = 0.985. The calculations of pore size distributions (PSD) followed the Barrett, Joyner and Halenda (BJH) procedure [56]. The pore diameters (D*_BJH_*) were estimated from the PSD maxima.

The microspheres were imaged using a scanning electron microscope (SEM) Duall Beam^TM^, Quanta3D FEG (Fei Company, Hillsboro, OR, USA) whereas the analysis of the size and distribution of the microspheres was made using Mastersized Analyser 2000 (Malvern, Instruments Ltd., Worcestershire, UK). The statistic of the distribution was calculated using the derived diameters in accordance with British standard BS2955:1993. According to this standard, *D*(0.1) is the size in microns of particle below which 10% of the sample lies; *D*(0.9) is the size of the particle below which 90% of the sample lies. *D*(0.5) refers to Mass Median Diameters (MMD), which is the size in microns at which 50% of the sample is smaller and 50% is larger. Span (the width of the size distribution) was calculated as

(1) $$span=\frac{D(0.9)-D(0.1)}{D(0.5)}$$

Thermogravimetry (TG) and differential scanning calorimetry (DSC) were performed with a Netzsch STA 449 F1 Jupiter thermal analyzer (Germany) in the range of 30 to 750 °C in helium or synthetic air (flow = 20 mL/min), at the heating rate of 10 °C min^−1^. All measurements were done in Al_2_O_3_ crucibles (mass of ~160 mg). As a reference, an empty Al_2_O_3_ crucible was applied. Sample masses of ~10 mg were used. From the TG curves the temperatures of 5 (*T*_5%_), 20 (*T*_20%_) and 50% (*T*_50%_) mass losses were determined. In turn, the temperature at which the maximum rate of mass loss (*T*_max_) for particular decomposition stages occurred was designed on the basis of differential TG (DTG) curves. The composition of the gas that evolved during the decomposition process was analyzed by a Bruker Tensor 27 FTIR spectrometer (Germany) coupled online to a Netzsch STA instrument by the Teflon transfer line with a 2 mm diameter heated to 200 °C. The FTIR spectra were recorded in the 600–4000 cm^−1^ spectral range with 16 scans per spectrum at 4 cm^−1^ resolution.

In order to obtain more detailed DSC data, the measurements were additionally carried out with a Netzsch 204 calorimeter (Selb, Germany) operating in a linear programmed mode. The measurements were conducted at a constant heating rate (10 °C/min) from room temperature to 500 °C in argon (flow = 20 mL/min) Measurements were taken in aluminum pans with a pierced lid (mass of ~40 mg). As a reference, an empty aluminum crucible was used. Sample masses were ~7.5 mg.

The polarity of the studied copolymers was determined according to the procedure proposed by Rohrscheider and modified by McReynolds [57]. The method is based on the measurement of the retention times of the test substances: benzene (*x*), butan-1-ol (*y*), pentan-2-one (*z*) and calculation of the retention indices *(I_x_*, *I_y_*, *I_z_* respectively) from the equation proposed by Kovats [58,59]:

(2) $$I_{x}=100\left[\frac{\log t_{R,x}^{\prime }-\log t_{R,n}^{\prime }}{\log t_{R,n+1}^{\prime }-\log t_{R,n}^{\prime }}+n\right]$$

where: $t_{R,x}^{\prime }\;$is the reduced retention time of the substance; $t_{R,n}^{\prime }\;$is the reduced retention time of the homologous alkane with the nearest shortest retention time; $t_{R,n+1}^{\prime }\;$is the reduced retention time of the next higher homologue eluted after homologue *n*; and *n* is the number of carbon atoms in the alkane molecule. The remaining indices (*I_y_* and *I_z_*) were calculated analogically.

Retention times for the test compounds were determined for the columns packed with the studied porous microspheres using Dani GC 1000 gas chromatograph (Milan, Italy) equipped with an injector and a thermal conductivity detector using stainless-steel columns (100 cm × 1.6 mm I.D.), and helium as carrier gas at a flow-rate of 50 mL/min. The samples were manually injected using a 1 µL syringe (SGE, North Melbourne, Australia). After determination the retention indices onto the studied copolymers, the McReynolds’ constants (Δ*I*) were obtained by calculating the difference between graphitized thermally carbon black (GTCB) and the Kovats’ index for benzene, butan-1-ol, and pentan-2-one in the examined stationary phase. The sum of the three calculated McReynolds’ constants was used to define the overall polarity of the phase under study. The measurements were carried out at 140 °C.

## 3. Results

**Figure 1:**
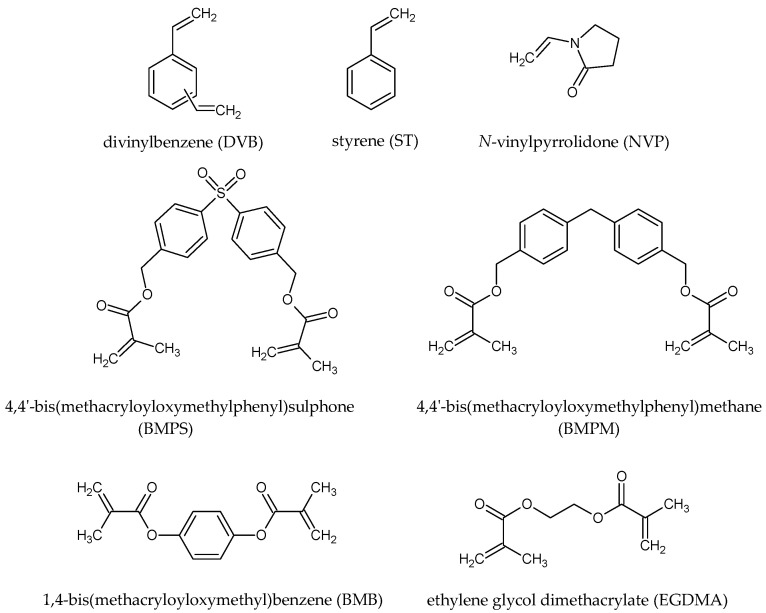
Chemical structures of monomers used for the synthesis of copolymeric microspheres.

Chemically diversified copolymers of DVB were synthesized via suspension polymerization. This method is especially suitable for the preparation of highly cross-linked, porous materials as regular microspheres having a diameter in the micrometric range. In the copolymer syntheses, various monomers that noticeably differed in chemical structure were used. We applied compounds that were both commercially available (St, DVB, VP, EGDMA) and synthesized in our laboratory (BMB, BMPS, BMPM). Figure 1 displays the chemical structure of all the used monomers.

**Figure 2:**
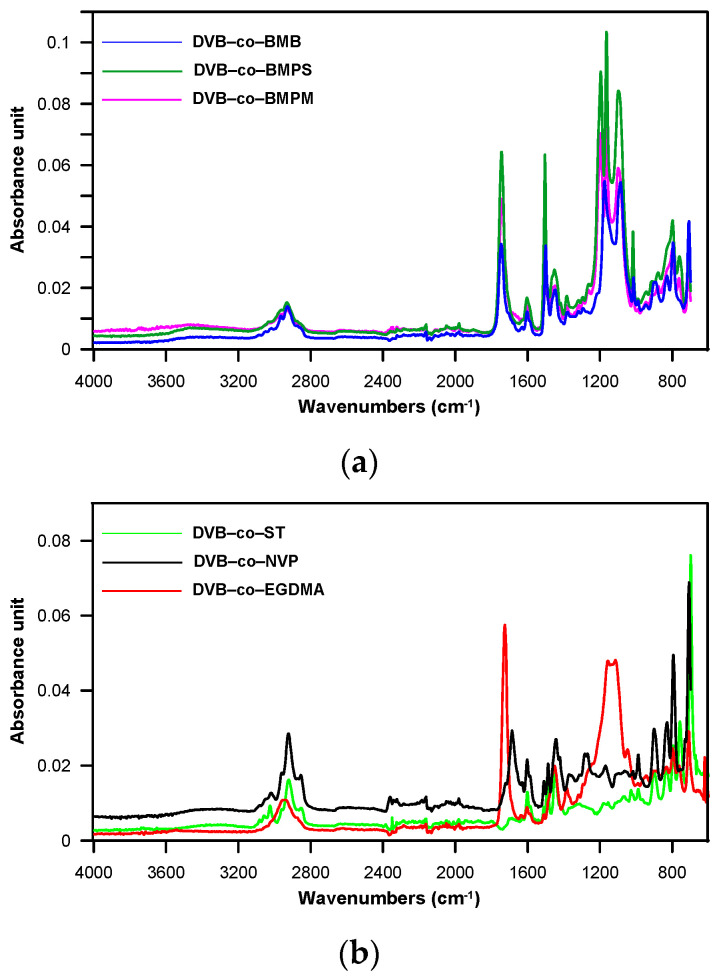
ATR-FTIR spectra of copolymeric microspheres. (**a**); DVB-*co*-BMB, DVB-*co*-BMPS and DVB-*co*-BMPM (**b**) DVB-*co*-ST, DVB-*co*-NVP and DVB-*co*-EGDMA.

The assumed chemical structure of the obtained copolymers was confirmed by ATR-FTIR analysis (see Figure 2). The spectra of all copolymeric beads exhibited bands indicating the existence of a benzene ring, i.e., at 3058–3018 cm^−1^ (*ν* C–H), 1604–1602 cm^−1^ and 1510–1492 cm^−1^ (*ν* C–C), 758 and 698 cm^−1^ (δ oop C–H of monosubstituted benzene), and 800–799 cm^−1^ (δ out-of-plane C–H of *p*-disubstituted benzene) and alkyl and alkylene groups, i.e., at 2962–2853 cm^−1^ (*ν* asym. and sym. C–H). The bands at 1452–1449 cm^−1^ originated from the vibrations of alkyl and alkylene groups as well as from the benzene ring. In the case of NVP-*co*-DVB copolymer, the spectrum also showed a band at 1686 cm^−1^ associated with the C=O stretching vibrations of the γ-lactam ring. On the other hand, for copolymers containing the ester groups, the carbonyl band could be seen at higher wave numbers (1747–1726 cm^−1^). The presence of the ester group was also manifested by the band at 1196–1087 cm^−1^ (ν C–O). A very low intense absorption band at 1638–1630 cm^−1^ coming from the unreacted C=C double bonds was also visible in some spectra.

The main drawback of the suspension polymerization is a broad-sized distribution of the obtained microspheres. Average size and size distribution depend on a number of factors including the diameter of the flask, the diameter of the stirrer, the volume ratio of the organic phase to the water phase, the mixing speed and the stabilizer concentration. While maintaining the constant value of the above-mentioned parameters, differences in the diameter of the synthesized microspheres were the result of the different chemical structure of the monomers. Because the monomers were chemically diversified, we dealt with different densities in the organic phase and, consequently, different interfacial tensions. As a result, microspheres of various diameters were created (Table 2).

**Figure 3:**
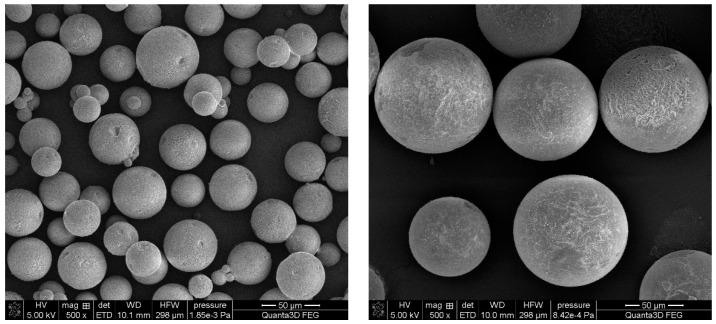
SEM images of DVB-*co*-ST (**left**) and DVB-*co*-EGDMA (**right**) copolymeric microspheres.

The mass median diameter of the synthesized microspheres was in the 54–155 µm range. Microspheres with the smallest diameter were obtained for the DVB-*co*-ST copolymer. What is important, is that this copolymer also indicated a broad-sized distribution. As a consequence, the value of span was the highest among all tested copolymers. The remaining copolymers were more uniform. The smallest value of the width of the size distribution (0.470) was observed for the DVB-*co*-EGDMA microspheres. These considerable differences in size distribution are clearly visible in Figure 3. The microsphere diameters determined on the basis of SEM images for all of the investigated copolymers were in the range of about 8 to 200 µm.

The deliberate aim of this work was to synthesize microspheres having a highly developed internal structure. Generally, the porous structure of the polymers was predominantly determined by phase separation during polymerization. At a given degree of conversion, a homogeneous polymerization mixture was separated into two phases: a solid, cross-linked polymer network, and one consisting of the solvent and unreacted comonomer liquid phase. The process was determined by the increase in the degree of crosslinking [*ν*-syneresis] or the change in polymer–solvent interactions [*χ*-syneresis]. In the case of a large amount of cross-linkers, phase separation occurred late and showed greater monomer-to-polymer conversion. As a result, a network of interconnected individual microglobules was formed characterized by a high surface area and pore-sized distribution with a maximum in the region of micro- to mesopores. To obtain such materials, monomers with a high functionality were used. Of all the applied substrates, only two possessed functionality equal to two (ST and NVP). The functionality of the remaining monomers was equal to four. Such a selection of monomers made possible the induction of late-phase separation and consequently the creation of a highly developed internal structure. Moreover, the choice of diluents strongly influenced the porous structure of the polymeric beads. If the diluent was a good solvent for the polymer (had good compatibility with the polymer network), phase separation took place later with a greater conversion of monomers to polymers. For this reason a polymer matrix with a high surface area was created. In the synthesis of the porous microspheres, toluene and chlorobenzene were used as porogenic solvents. Generally, these control porous properties through the solvation of the polymer chains in the reaction medium during the early stages of the polymerization. Because the Hildebrand solubility parameter of toluene is equal to 18.2 (MPa)^0.5^ its solvating power for most of the synthesized copolymers was quite high. As a result, copolymers with a remarkably developed internal structure were obtained. The value of the specific surface area of copolymers synthesized in the presence of toluene changed from 417 to 746 m^2^/g (Table 3).

**Figure 4:**
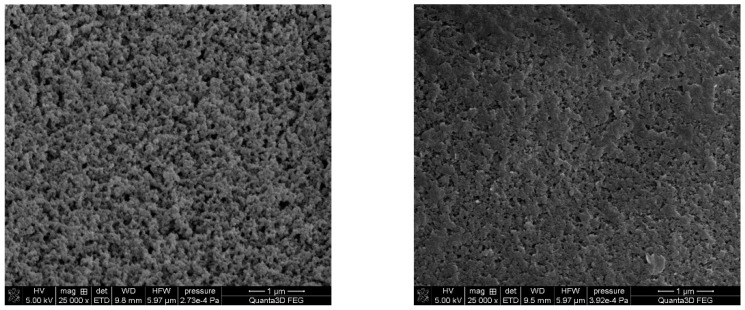
SEM microphotographs of the surface of DVB-*co*-NVP (**left**) and DVB-*co*-BMPS (**right**) copolymers.

In the case of DVB-*co*-BMPS and DVB-*co*-BMPM copolymers, the initial test with toluene as the solvent resulted in low specific surface areas (below 100 m^2^/g). That is why we decided to use the more harmful chlorobenzene with the Hildebrand solubility parameter equal to 19.6 (MPa)^0.5^. The use of chlorobenzene as a porogenic solvent made it possible to obtain copolymers with a much developed internal structure. Figure 4 displays exemplary SEM microphotographs of copolymers with distinctive specific surface areas.

On the basis of TG studies it can be stated that all the studied microspheres were characterized by a remarkable thermal resistance for polymers, both in inert and oxidative atmospheres. In helium, the *T*_5%_ determined for all microspheres was above 300 °C: the highest value (346 °C) was shown by DVB-*co*-NVP copolymer and the lowest (302 °C) by DVB-*co*-ST copolymer (Table 4). Generally, cross-linked polymers synthesized from bifunctional monomers do not possess such a high thermal stability. The high thermal resistance of DVB-*co*-NVP copolymer could have resulted from the fact that not all of the NVP used in the synthesis was incorporated in the polymer network; consequently, the final material had poly(DVB) regions of a high thermal stability. The remaining copolymers based on tetrafunctional monomers showed *T*_5%_ in the 321–339 °C range. Among these copolymers, the least stable was the one based on the aliphatic monomer EGDMA. In general, this tendency followed the same pattern as for the other indicators. The values of *T*_20%_ ranged from 336 to 389 °C, whereas *T*_50%_ ranged from 374 to 484 °C (Table 4).

**Figure 5:**
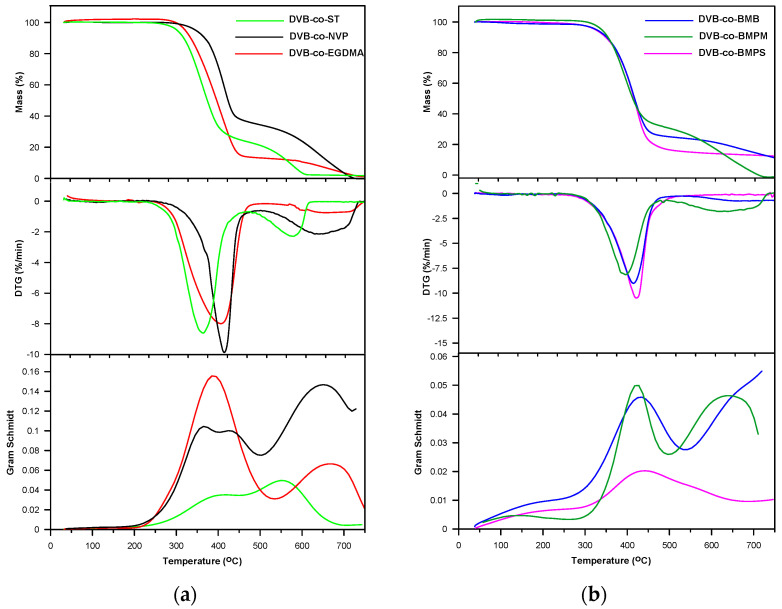
TG, DTG and Gram–Schmidt curves of copolymers obtained under helium atmosphere. (**a**); DVB-*co*-ST, DVB-*co*-NVP and DVB-*co*-EGDMA; (**b**) DVB-*co*-BMB, DVB-*co*-BMPS and DVB-*co*-BMPM.

Based on the data collected in an inert atmosphere, it could also be seen that in the case of copolymers synthesized with the use of ST, NVP, EGDMA, BMB and BMPM, the degradation process was conducted in two stages. However, the main mass loss was observed in the first one. The highest value of mass loss (89%) was observed for the copolymer where EGDMA was used as the aliphatic comonomer. In the case of the DVB-*co*-BMPS copolymer, the second step was not observed in the measured temperature range. As a consequence, quite a high residual mass was noticed (14.0%). Figure 5 presents the TG and DTG curves obtained in helium for all copolymeric microspheres. In the first step, with a maximum at 346–421 °C, it is the functional groups that were mainly subjected to thermal degradation. Generally, the decomposition of methacrylate derivatives began with the degradation of the ester groups. Among the copolymers under study, four were methacrylate derivatives. From the literature, it follows that such materials can decompose according to the α- and β-hydrogen bond scission. The products of the α-hydrogen bond scission are mainly aldehydes and carboxylic acids, while from the β-hydrogen bond scission vinyl compounds are created [60].

**Figure 6:**
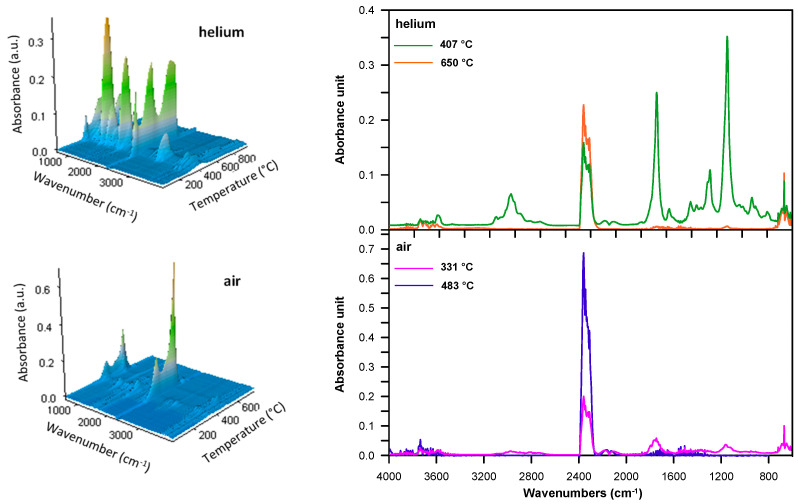
FTIR spectra of volatile products evolved during the thermal decomposition of DVB-*co*-EGDMA copolymer: 3D (**left**) and extracted at the maxima of decomposition (**right**).

When all copolymers were methacrylate derivatives, the FTIR spectra obtained at the first decomposition maximum showed absorption bands indicating the creation of both vinyl compounds (at ~910 and 990 cm^−1^ and at 3095–3078 cm^−1^ attributed to the C–H out-of-plane deformation and stretching vibrations, respectively) and carbonyl ones (at 1772–1770 cm^−1^, connected with the C=O stretching vibrations), including aldehydes and esters. The existence of aldehydes was manifested by the bands at ~2820 and 2720 cm^−1^, which were associated with the C–H stretching vibrations; however, the band at ~1125 cm^−1^, characteristic of C–O stretching vibrations, was a sign that esters were present. Moreover, bands typical of carbon dioxide (at 2359–2310 cm^−1^, attributed to asymmetric stretching vibrations and at 669 cm^−1^, caused by degenerate bending vibrations), carbon monoxide (at 2181 and 2114 cm^−1^, related to stretching vibrations) and water (at ~4000–3500 cm^−1^, connected with stretching vibrations and at ~1800–1300 cm^−1^ with bending vibrations) were visible. Figure 6 illustrates an example of the FTIR spectra of volatile products evolved during the thermal decomposition of DVB-*co*-EGDMA copolymer in the whole temperature range of the measurement (3D spectrum) and the temperatures of the maximum rate of mass loss.

**Figure 7:**
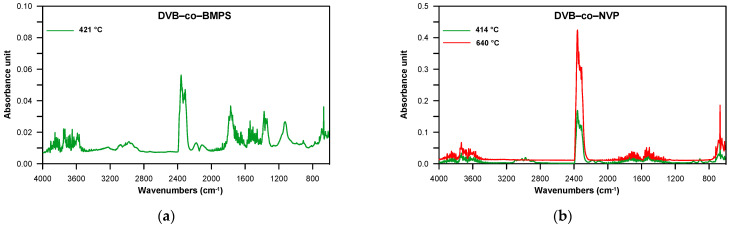
FTIR spectra of volatile products evolved during the thermal decomposition of DVB-*co*-BMPS and DVB-*co*-NVP copolymers collected at the maxima of decomposition in helium atmosphere. (**a**) DVB-*co*-BMPS; (**b**) DVB-*co*-NVP.

The DVB-*co*-BMPS copolymer, apart from methacrylate groups, also contained the diphenyl sulfone unit. As a consequence, additional bands pointing to the creation of sulfur dioxide associated with stretching vibrations at 1375–1340 cm^−1^ were detected in the FTIR spectra, which is clearly visible in Figure 7. In turn, the spectra received for DVB-*co*-ST and DVB-*co*-NVP copolymers revealed bands connected with the evolution of carbon dioxide, carbon monoxide and water as well unsaturated products: for example, for DVB-*co*-NVP (Figure 7). In the case of all the studied polymers, random chain scission took place as well. It should also be noted that in the first step, the decomposition of cross-linked parts of copolymers also started. Nevertheless, the second step, which had a maximum at 539–650 °C, was mostly attributable to the degradation of the highly cross-linked units (primary nuclei) created during very early phase separation in the polymerization reaction. The detected volatile decomposition products were carbon dioxide and water (see FTIR spectra received for DVB-*co*-EGDMA in Figure 6). Furthermore, the emission maxima of the detected gases on the Gram–Schmidt plots were in relatively good accordance with the maxima on the DTG curves (Figure 5).

**Figure 8:**
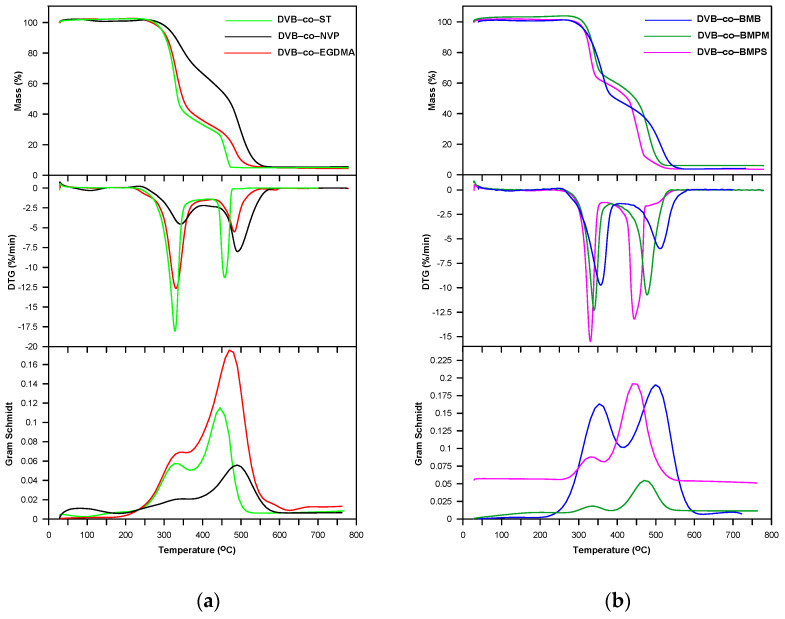
TG, DTG and Gram–Schmidt curves of copolymers obtained under air atmosphere. (**a**); DVB-*co*-ST, DVB-*co*-NVP and DVB-*co*-EGDMA; (**b**) DVB-*co*-BMB, DVB-*co*-BMPS and DVB-*co*-BMPM.

Regarding oxidative conditions, one can say that the studied copolymers exhibited a somewhat worse thermal stability (*T*_5%_: 294–320 °C and *T*_20%_: 316–354 °C) compared to the inert ones (Table 5). Their degradation occurred in two partially superimposed steps with maxima at 328–357 °C and 444–500 °C as shown in Figure 8.

During the decomposition of all the copolymers in synthetic air, practically the same types of volatile substances as in helium atmosphere were released in both the first and second steps. The difference is that carbon monoxide was also detected in the second (see FTIR spectra for DVB-*co*-EGDMA copolymer in Figure 6). As for the DVB-*co*-BMPS copolymer decomposition, the FTIR spectra collected in the second step demonstrated bands originating from carbon dioxide, carbon monoxide, water and sulfur dioxide. The analysis of FTIR spectra also showed that a much greater amount of carbon dioxide had been emitted in the second stage of copolymer decomposition. This was due to the intense oxidation of the products obtained in the first stage.

**Figure 9:**
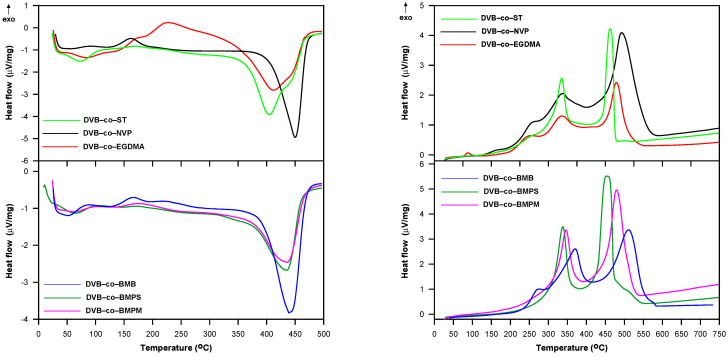
DSC curves of copolymers obtained in helium (**left**) and air (**right**) atmosphere.

To gain more comprehensive information about the thermal behavior of the studied copolymers, further DSC measurements were carried out. Figure 9 displays the DSC curves obtained in helium and air atmospheres. For the curves obtained in helium, both exothermic and endothermic peaks are visible. The first endothermic peak is connected with the evaporation of moisture from the internal structure of the copolymers. All samples were dried in vacuo before experiments. However, as they were highly porous and chemically diversified they easily adsorbed moisture from the atmosphere during weighting. Interestingly, the presence of adsorbed water in the samples is practically invisible on the TG/DTG curves. For most samples, the first endothermic peak was followed by an exothermic one that could be attributed to the post cross-linking of unreacted double bonds still present in the polymer matrix. Only the DVB-*co*-ST copolymer exhibited no exothermic peaks. which indicated a high degree of double-bond conversion. However, this polymer started to decompose at the lowest temperature of all investigated materials. The decomposition process was represented by the second endothermic peak with a maximum in the 405–450 °C range.

On the other hand, only exothermic events were manifested on DSC curves received in the air atmosphere. At the beginning, the oxidation of unreacted double bounds occurred. This process was especially visible for DVB-*co*-BMB, DVB-*co*-NVP and DVB-*co*-EGDMA copolymers, which indicated a considerable amount of double bonds remaining in these materials. The second peaks with a maximum above 300 °C pointed to the thermal degradation of functional groups and linear fragments of the polymeric structure. Above 450 °C, the oxidation reaction of the cross-linked residues took place, which was confirmed by the FTIR spectra of emitted gases. The main detected gases were carbon dioxide and water.

The high thermal stability of the studied copolymers allowed inverse gas chromatography to be applied for further characterization. Table 6 presents Kovats’ retention indices for the McReynolds’ test substances determined at 140 °C. On their basis, overall polarity was assessed and a polarity index (ΣΔ*I*) was calculated. A polarity index ranks adsorbents according to the sum of the McReynolds’ constant of the test substances. As a rule, the higher the polarity index, the more polar the adsorbent. As expected, the lowest value of ΣΔ*I* (315) was observed for the DVB-*co*-ST copolymer, which did not contain a functional group. The incorporation of even an inconsiderable amount of a methacrylate group into a polymer matrix resulted in an increase of the overall polarity value to 357 as could be seen for DVB-*co*-BMB. The equimolar reaction of DVB with aliphatic EGDMA gave a material with polarity exceeding 400, whereas the aromatic BMPM generated a further increase of polarity to 494. The additional presence of the sulfone group led to the highest value of overall polarity (518) among all studied copolymers. Worth noticing is that materials with a high polarity (510) could be also obtained by applying VP as a functional monomer.

## 4. Conclusions

Permanently porous copolymers of DVB with diverse functional monomers in the form of regular microspheres were successfully synthesized via suspension polymerization. As intended, they possess a highly developed internal structure and remarkable thermal stability. Depending on the monomer used, microspheres with a specific surface area in the 418–746 m^2^/g range were synthesized. Importantly, all copolymers with various functional groups were characterized by a higher surface-area value than that of the DVB-*co*-ST copolymer. Furthermore, the incorporation of the functional groups did not negatively affect the thermal behavior of the materials. All of the obtained copolymers showed good thermal stability. In helium, their temperatures of 5% mass losses were over 300 °C, whereas in air these values were only slightly lower. Generally, their decomposition in both atmospheres took place in two partially overlapping stages and was accompanied by the evolution of the same kinds of volatile products. The basic volatile products were vinyl compounds, carbon dioxide, carbon monoxide, and water. Moreover, aldehydes and esters were detected for microspheres containing methacrylate groups in the structure; additionally, sulfur dioxide was detected for the DVB-*co*-BMPS copolymer. It should be stressed that the presence of the functional groups contributed to the increase in overall polarity in comparison with the DVB-*co*-ST copolymer.

## Figures and Tables

**Table 1:** Designations and experimental parameters of copolymeric microspheres synthesis.

Copolymeric Microsphere	Monomers		Monomers Ratio	Diluents (mL)	
				Toluene	Chlorobenzene
DVB-*co*-ST	DVB	ST	1:1	22.5	-
DVB-*co*-NVP		NVP	1:1	22.5	-
DVB-*co*-EGDMA		EGDMA	1:1	22.5	-
DVB-*co*-BMB		BMB	3:1	22.5	-
DVB-*co*-BMPS		BMPS	1:1	-	22.5
DVB-*co*-BMPM		BMPM	1:1	-	22.5

**Table 2:** The statistical data of particle size distribution.

Copolymeric Microsphere	*D*(0.1)	*D*(0.5)	*D*(0.9)	Span
DVB-*co*-ST	21	54	86	1.203
DVB-*co*-NVP	51	89	121	0.786
DVB-*co*-EGDMA	76	102	124	0.470
DVB-*co*-BMB	38	78	98	0.769
DVB-*co*-BMPS	84	142	178	0.662
DVB-*co*-BMPM	86	155	196	0.710

**Table 3:** The main parameters of the porous structure of the copolymeric microspheres.

Copolymeric Microsphere	Specific Surface Area *S*_BET_ (m^2^/g)	Pore Volume *V* (cm^3^/g)	Pore Diameter *D*_BJH_ (Å)
DVB-*co*-ST	418	0.440	50
DVB-*co*-NVP	746	1.045	100
DVB-*co*-EGDMA	417	0.801	158
DVB-*co*-BMB	712	1.219	240
DVB-*co*-BMPS	416	0.693	240
DVB-*co*-BMPM	512	0.478	185

**Table 4:** TG and DTG data of the copolymeric microspheres obtained in helium atmosphere.

Copolymeric Microsphere	*T*_5%_ (°C)	*T*_20%_ (°C)	*T*_50%_ (°C)	*T*_max1_ (°C)	*T*_max2_ (°C)	Residual Mass (%)
DVB-*co*-ST	302	336	374	364	578	1.5
DVB-*co*-NVP	346	389	423	414	640	0.5
DVB-*co*-EGDMA	321	354	397	407	650	1.0
DVB-*co*-BMB	327	377	418	415	660	8.7
DVB-*co*-BMPS	324	376	416	421		14.0
DVB-*co*-BMPM	339	373	413	396	615	0.0

**Table 5:** TG and DTG data of the copolymeric microspheres obtained in synthetic air atmosphere.

Copolymeric Microsphere	*T*_5%_ (°C)	*T*_20%_ (°C)	*T*_50%_ (°C)	*T*_max1_ (°C)	*T*_max2_ (°C)	Residual Mass (%)
DVB-*co*-ST	294	316	336	328	458	5.0
DVB-*co*-NVP	320	354	466	343	491	5.5
DVB-*co*-EGDMA	295	321	350	331	483	4.5
DVB-*co*-BMB	314	344	395	357	500	4.2
DVB-*co*-BMPS	313	329	426	330	444	3.7
DVB-*co*-BMPM	317	340	446	340	478	6.0

**Table 6:** Kovats’ retention indices for the McReynolds’ test substances and overall polarity (ΣΔ*I*) for the porous copolymeric microspheres.

Copolymeric Microsphere	Kovats’ Retention Indices			McReynolds’ Constants			
	*I_x_* (Benzene)	*I_y_* (Butan-1-ol)	*I_z_* (Pentan-2-one)	Δ*I_x_*	Δ*I_y_*	Δ*I_z_*	ΣΔ*I*
DVB-*co*-ST	619	646	678	45	157	113	315
DVB-*co*-NVP	631	755	751	58	266	186	510
DVB-*co*-EGDMA	647	698	721	73	209	156	438
DVB-*co*-BMB	623	663	699	49	174	134	357
DVB-*co*-BMPS	669	718	759	95	229	194	518
DVB-*co*-BMPM	648	730	753	74	232	188	494
GTCB	574	489	565	-	-	-	-

## Data Availability

The data presented in this study are available on request from the corresponding authors.
